# Potassium citrate prevents increased osteoclastogenesis resulting from acidic conditions: Implication for the treatment of postmenopausal bone loss

**DOI:** 10.1371/journal.pone.0181230

**Published:** 2017-07-17

**Authors:** Donatella Granchi, Elena Torreggiani, Annamaria Massa, Renata Caudarella, Gemma Di Pompo, Nicola Baldini

**Affiliations:** 1 Orthopedic Pathophysiology and Regenerative Medicine Unit, Rizzoli Orthopedic Institute, Bologna, Italy; 2 Villalba Hospital, GVM Care and Research, Bologna, Italy; 3 Department of Biomedical and Neuromotor Sciences, University of Bologna, Bologna, Italy; Charles P. Darby Children's Research Institute, 173 Ashley Avenue, Charleston, SC 29425, UNITED STATES

## Abstract

The extracellular acidic milieu in bones results in activation of osteoclasts (OC) and inhibition of osteoblasts (OB) causing a net loss of calcium from the skeleton and the deterioration of bone microarchitecture. Alkalinization through supplementation with potassium citrate (K citrate) has been proposed to limit the osteopenia progression, even though its pharmacological activity in bone microenvironment is not well defined. We evaluated if K citrate was able to prevent the adverse effects that acidic milieu induces on bone cells. OC and OB were maintained in neutral (pH 7.4) versus acidic (pH 6.9) culture medium, and treated with different K citrate concentrations. We evaluated the OC differentiation at seven days, by counting of multinucleated cells expressing tartrate-resistant acid phosphatase, and the activity of mature OC at 14 days, by quantifying of collagen degradation. To evaluate the effects on OB, we analyzed proliferation, mineralization, and expression of bone-related genes. We found that the low pH increased OC differentiation and activity and decreased OB function. The osteoclastogenesis was also promoted by RANKL concentrations ineffective at pH 7.4. Non-cytotoxic K citrate concentrations were not sufficient to steadily neutralize the acidic medium, but a) inhibited the osteoclastogenesis, the collagen degradation, and the expression of genes involved in RANKL-mediated OC differentiation, b) enhanced OB proliferation and alkaline phosphatase expression, whereas it did not affect the in vitro mineralization, and c) were effective also in OC cultures resistant to alendronate, i.e. the positive control of osteoclastogenesis inhibition. In conclusion, K citrate prevents the increase in OC activity induced by the acidic microenvironment, and the effect does not depend exclusively on its alkalizing capacity. These data provide the biological basis for the use of K citrate in preventing the osteopenia progression resulting from low-grade acidosis.

## Introduction

The deterioration of bone microarchitecture is a common event in postmenopausal women with the consequent decrease in bone mass and increased susceptibility to fracture [1]. The bone loss starts early and continues for many years depending on many causes, all noxious to the skeletal homeostasis since impair equilibrium between the removal of old bone by osteoclasts (OC) and the formation of bone matrix by osteoblasts (OB) [2]. An imbalance between these processes, due to enhanced osteoclastogenesis or excessive OC activity, is implicated in several conditions characterized by the loss of bone mass and compromised bone strength, including osteopenia and osteoporosis [3]. It is well recognized that skeletal homeostasis influences a variety of physiological functions, but the regulation of acid-base balance is undoubtedly one of the most important [1]. The skeleton is an alkaline reservoir that is able to buffer systemic acidosis by modifying the composition of the bone mineral matrix, namely hydroxyapatite [4]. In physiological conditions, the pH values of venous and arterial blood fluctuate from 7.36 to 7.40, respectively [5]. Postmenopausal estrogen deficiency [6], diets rich in salt and meat proteins [7], the decreased renal function due to aging [8], are some of the conditions in which a high acid loading may exceed the physiological neutralization capacity, eventually leading to a latent or low-grade acidosis [4]. The pH decrease can also arise locally in the bone tissue. Normal intra-bone pH has been determined in blood samples from experimental models and differs from metaphysis to bone marrow, i.e. from 7.3 to 7.4 [9], but lower values are expected in the interstitial fluid around bone cells [10]. Even though bone is a well-perfused tissue, the amount of blood supply tends to decline with age, thus promoting an hypoxic status [11]. The poor vascularization leads to an increase in CO_2_ and an excess of protons derived from cellular metabolism that accumulates in the tissue and decreases the interstitial pH [12].

A low extracellular pH favors the resorption of the mineralized matrix that provides hydroxyl groups for opposing acid loading when it overcomes the physiological neutralization capacity [4]. Actually, the higher the acidosis, the higher the bone demineralization and the loss of calcium from bones. *In vitro* experiments have shown that acidosis directly influences the cellular component of bone. In fact, OC are virtually inactive at neutral pH (pH 7.4), but the resorption activity increases dramatically when pH drops below 6.9. On the contrary, acidosis significantly inhibits the osteogenic function of OB, including the production of extracellular matrix, the activity of alkaline phosphatase, and the formation of trabecular bone [4].

Since the low pH is a risk factor that accelerates bone loss, the use of measures to counteract an acid overload may have beneficial effects in preventing osteopenia progression, e.g., in postmenopausal women, or in supporting conventional therapy for osteoporosis. To this purpose, some authors have suggested the use of proton pump inhibitors [13], but the long-term treatment with these drugs seems to inhibit calcium absorption and, in turn, increase the fracture risk [14]. As an alternative, supplementation with alkalizing compounds has been suggested as a tool to limit the detrimental effect of acidosis and prevent bone loss [15]. Potassium citrate (K citrate) is a potassium salt of the citric acid that is used to increase the urinary pH, thus preventing the precipitation of solutes and the formation of kidney stones [16]. However, recent findings demonstrated that lithogenic risk factors are detectable also in postmenopausal women who exhibit osteopenia, but not urolithiasis, thus reinforcing the hypothesis that potassium citrate supplementation could have beneficial effects in preventing the bone loss progression [17]. Clinical data regarding the effectiveness of K citrate on calcium metabolism are encouraging [18–22], but it is still unclear whether the beneficial effects are exclusively due to its alkalizing ability or if it may also influence bone cell activity.

In this study, we investigated if K citrate is able to prevent the adverse effects that acidic milieu induces on primary human bone cells. We aimed to search for the biological basis that may support the use of K citrate for preventing the bone loss following a low-grade acidosis.

## Materials and methods

### Preparation of culture media and pH measurement

Dulbecco׳s Modified Eagle׳s Medium (DMEM), Low Glucose-DMEM, and α-minimum essential medium (αMEM) (Sigma-Aldrich, St.Louis, MO) were maintained at pH 6.9 or 7.4 by using different concentrations of NaHCO_3_, according to the Henderson-Hasselbach equation. Complete culture media contained 10% fetal bovine serum (FBS) (Sigma-Aldrich), 20 mM glutamine (Gibco, Monza, Italy), penicillin (100 U/mL) and streptomycin (100 μg/mL) (Thermo Fisher Scientific, Waltham, MA, USA). Differentiation medium for OC or RAW 264.7 cultures was complete DMEM containing 25% of a cell line supernatant containing pro-osteoclastogenic factors (human neuroblastoma SH-SY5Y (ATCC, CRL-2266) [23]. For selected experiments, complete DMEM was added with RANKL (0.5, 5 and 50 ng/mL) and M-CSF (10 ng/mL) (Peprotech, London, UK). Osteogenic medium for the mineralization assay was complete αMEM supplemented with 50 μM L-ascorbic acid 2-phosphate, 10^−8^ M dexamethasone, and 10 mM β-glycerophosphate (Sigma-Aldrich). The stock solution of K citrate (30 mM) was obtained dissolving the powder (C_6_H_5_K_3_O_7_*H_2_O, Gadot Biochemical Industries Ltd, Haifa Bay, Israel) in complete DMEM at pH 6.9. The pH measurement of K citrate dilution was performed on samples prepared as for the cell cultures (see below) and maintained at 37°C in a humidified atmosphere of 5% CO_2_. The pH of the culture media was measured before each experiment by a digital pH-meter (6230N, Jenco, San Diego, CA, USA).

### Cytotoxicity screening

Cytotoxicity was evaluated on RAW 264.7 cell line and human OB. The RAW 264.7 cell line (American Type Culture Collection; ATCC TIB-71) was maintained in complete DMEM, at 37°C in a humidified atmosphere of 5% CO_2_. Cells were seeded in 8-well chamber slides (0.81 cm^2^/well) at the density of 7x10^4^ cells/cm^2^ in 0.4 mL of culture medium.

After 24 hours, the culture medium was discarded and replaced with 0.4 mL of complete DMEM at pH 6.9 containing K citrate (from 0.03 to 40 mM). After 96 hours, cells were exposed to 10% Alamar Blue (Invitrogen, Monza, Italy), and after 4 hours the fluorescence was read at a wavelength of 535–590 nm using a microplate-reader (Infinite F200pro, Tecan, Milan, Italy). OB cultures were obtained from trabecular bone chips of three subjects undergoing primary total hip replacement, as previously described [24]. The tissue collection was approved by the Institutional Review Board of Rizzoli Orthopedic Institute, and a written informed consent was obtained from patients. After reaching the confluence, cells were detached by trypsin and samples were pooled together to reduce the effect of individual variability. OB were seeded at the density of 1x10^4^ cells/cm^2^ and maintained at 37°C in a humidified atmosphere of 5% CO_2_ in complete DMEM at pH 7.4. After 24 hours, the culture medium was discarded and replaced with 1 mL of complete DMEM at pH 7.4 and 6.9 supplemented with K citrate (from 0.03 to 30 mM). The culture medium was changed every two days. Proliferation was evaluated after 96 hours by using the Alamar Blue assay as described previously. A dose-response curve was plotted, and the concentration giving 50% of growth inhibition (IC50) was calculated.

### Evaluation of OC formation and activity

OC cultures were derived from peripheral blood mononuclear cells (PBMC), as previously described [25]. PBMC were obtained from buffy-coats discarded from blood donations of eight volunteers (AVIS, Bologna, Italy). PBMC were plated in 8-well chamber slides (Thermo-Fisher Scientific, 0.81 cm^2^/well) at the density of 3×10^6^ cells/cm^2^ and incubated for 2 hours at 37°C in a humidified atmosphere of 5% CO_2_. After 2 hours, non-adherent cells were removed and PBMC were maintained in differentiation medium at pH 7.4 and 6.9. The acidic or the neutral (for selected experiments) differentiation medium was supplemented with K citrate from 0.03 to 30 mM. As a positive control, cells were treated with Alendronate 10^−5^ M (Ale 10^−5^) (Sigma-Aldrich). To evaluate the synergy between Ale and K citrate, OC cultures were treated with Ale 10^−5^ M and Ale 10^−6^ M, combined or not with K citrate 0.15 and 0.3 mM. Moreover, we evaluated the osteoclastogenesis using different concentrations of RANKL (0.5, 5 and 50 ng/mL) at acidic or physiological conditions. The culture medium was replaced every two days, and after 7 days the differentiation of PBMC into mature OC was evaluated by analyzing the expression of the tartrate-resistant acid phosphatase (TRAcP) (Acid Phosphatase, Leukocyte kit, Sigma-Aldrich), according to the manufacturer’s protocols. Then, cells were dark incubated with 2.25 μg/mL of Hoechst 33258 at room temperature for 10 minutes to highlight number and morphology of the nuclei, and visualized with a Nikon Eclipse E800M fluorescence microscope (Nikon, Tokyo, Japan). Only TRAcP positive cells with three or more nuclei were considered as OC. The quantification of OC formation was obtained by counting the total number of OC in 6 random optical fields.

After 7 days, the OC were fixed and stained with phalloidin–tetramethylrhodamine B isothiocyanate (TRITC) 0.3 μg/mL to highlight the F-actin ring structure in selected experiments.

Resorption activity by mature OC was measured by using the OsteoLyse bone resorption assay kit (Lonza Walkersville, Inc., Walkersville, MD, USA). PBMC were seeded onto the collagen-coated surface of a 96-wells microplate and cultured in OC differentiation medium at pH 7.4. After 7 days, the culture medium was discarded and mature OC were maintained at pH 7.4 or pH 6.9 with the addition of K citrate (0.3 and 1.5 mM) and alendronate 10^−5^ M. Following 2 and 7 days of treatment, 10 μl of supernatant were added to 200 μl of Fluorophore Releasing Reagent. The Relative Fluorescence Units (RFU) of each well was determined using the microplate-reader, with excitation at 340 nm and emission at 615 nm.

Finally, after 14 days, the expression of NFkB-related genes and OC genes were analyzed in duplicate by quantifying the transcripts by Real-Time Polymerization Chain Reaction (RT-PCR). RNA was extracted with NucleoSpin RNA II (Invitrogen) and reverse transcribed into cDNA with MuLV Reverse Transcriptase (Applied Biosystems, Foster City, CA), according to the manufacturer’s protocols. RT-PCR was performed by amplifying 1 ug of cDNA using the Light Cycler instrument and the Universal Probe Library system (Roche Applied Science, Monza, Italy). Probes and primers are reported in Table 1. The results were expressed as a ratio between the gene of interest and β-actin as reference gene according to the 2-ΔΔCT method [26].

**Table 1:** List of primers and probes.

Gene	Full name	Accession Number	Primers	Probe*
**ALPL**	Alkaline phosphatase, liver/bone/kidney	NM_000478.3	F = GGGTCAGCTCCACCACAA R = GCATTGGTGTTGTACGTCTTG	52
**β-Actin**	actin, beta	NM_001101.2	F = CCAACCGCGAGAAGATGA R = CCAGAGGCGTACAGGGATAG	64
**BGLAP**	bone gamma-carboxyglutamate protein (osteocalcin)	NM_199173.2	F = GGCGCTACCTGTATCAATGG R = TCAGCCAACTCGTCACAGTC	1
**CTSK**	cathepsin K	NM_000396.2	F = GGATATGTTACTCCTGTCAAAAATCA R = TGCCAGTTTTCTTCTTGAGTTG	37
**CD44**	CD44 molecule (Indian blood group)	NM_000610.3	F = TGACACATATTGCTTCAATGCTT R = TGGGCAGGTCTGTGACTG	39
**COL1A1**	Collagen Type I alpha 1 chain	NM_000088.3	F = CCCCTGGAAAGAATGGAGAT R = AATCCTCGAGCACCCTGA	60
**DC-STAMP**	dendrocyte expressed seven transmembrane protein	NM_030788.3	F = GACGGCTGAGTCTCTATCTTACAA R = AAGAGAATTGTTGGTGCGATG	73
**MMP-9**	matrix metallopeptidase 9	NM_004994.2	F = GAACCAATCTCACCGACAGG R = CCACCCGAGTGTAACCATA	6
**OPG**	Tumor necrosis factor receptor superfamily, member 11b (Osteoprotegerin)	NM_002546.2	F = GAAGGGCGCTACCTTGAGAT R = GCAAACTGTATTTCGCTCTGG	79
**Osterix**	Osterix	AF477981.1	F = CATCTGCCTGGCTCCTTG R = CAGGGGACTGGAGCCATA	69
**RANK**	receptor activator of nuclear factor-kappa B	AF018253.1	F = GCAGGTGGCTTTGCAGAT R = CACTGTCAGAGGTAGTGCATTTAG	25
**RANKL**	Receptor activator of nuclear factor kappa-B ligand	NM_003701.2	F = TGATTCATGTAGGAGAATTAAACAGG R = GATGTGCTGTGATCCAACGA	17
**RUNX2**	runt-related transcription factor 2, transcript variant 1 and variant 3	NM_001024630.2; NM_004348.3	F = GTGCCTAGGCGCATTTCA R = CACCTGCCTGGCTCTTCTTA	87
**rRNA 18S**	Human 18S ribosomal RNA	X03205.1	F = GCAATTATTCCCCATGAACG R = GGGACTTAATCAACGCAAGC	48

F: forward; R: reverse

* Probes selected by using web-based assay design software (ProbeFinderhttps://www.roche-applied-science.com)

### Evaluation of OB gene expression and mineralization

To evaluate the mineralization ability and the expression of genes useful to monitor the osteogenic potential, we used a pool of second passage OB cultures from three male donors that were selected on the basis of their osteogenic properties that have been previously described [27]. Confluent cells were incubated in osteogenic medium at pH 7.4 and 6.9 with the same K citrate concentrations used for the proliferation assay. The culture medium was changed twice a week. After 14 days, the expression of bone-related genes was analyzed in duplicate by quantifying the transcripts by Real-Time Polymerization Chain Reaction (RT-PCR). RNA was extracted with Trizol (Invitrogen) and reverse transcribed as described above. Probes and primers are reported in Table 1. The results were expressed as a ratio between the gene of interest and rRNA18s as reference gene according to the 2-ΔΔCT method [26].

After 21 days, calcium-phosphate (CaP) deposits were stained with 1% Alizarin Red S (Sigma-Aldrich), as previously described [24]. The CaP mineral nodules were counted using a Nikon Eclipse-TE 2000-S microscope. To quantify the Alizarin Red bound to the extracellular matrix, 500 μL of 10% cetylpyridinium chloride (p/v) (Sigma-Aldrich) were added to each well, and the plate was incubated at room temperature for 30 min with shaking. Duplicate aliquots of solubilized dye (200 μL) were transferred in 96-well plate and read in using the microplate-reader, with a 570 nm wavelength.

After 7 days, cytochemical alkaline phosphatase (ALP) analysis assay was performed directly on duplicate wells of culture plates using a commercial kit (Sigma).

### Statistical analysis

Statistical analysis was performed using StatView 5.01 for Windows (SAS Institute Inc, Cary, NC). Quantitative data were expressed as arithmetic mean plus or minus the standard error of the mean (SEM). Paired analyses were applied to highlight the significant differences due to the exposure of the cell cultures at pH 7.4 or pH 6.9. The one-way analysis of variance (ANOVA) was used to test the effects of the multiple independent variables (K citrate concentrations, alendronate) on the dependent variables, i.e. the biological activities of OC and OB. When ANOVA was positive, the *post hoc* Bonferroni-Dunn test was applied to highlight the differences among the subgroups. In addition, P values ≤ 0.05 were considered as statistically significant.

## Results

### Non-cytotoxic concentrations of K citrate are not sufficient to neutralize the acidic culture medium.

**Fig 1:**
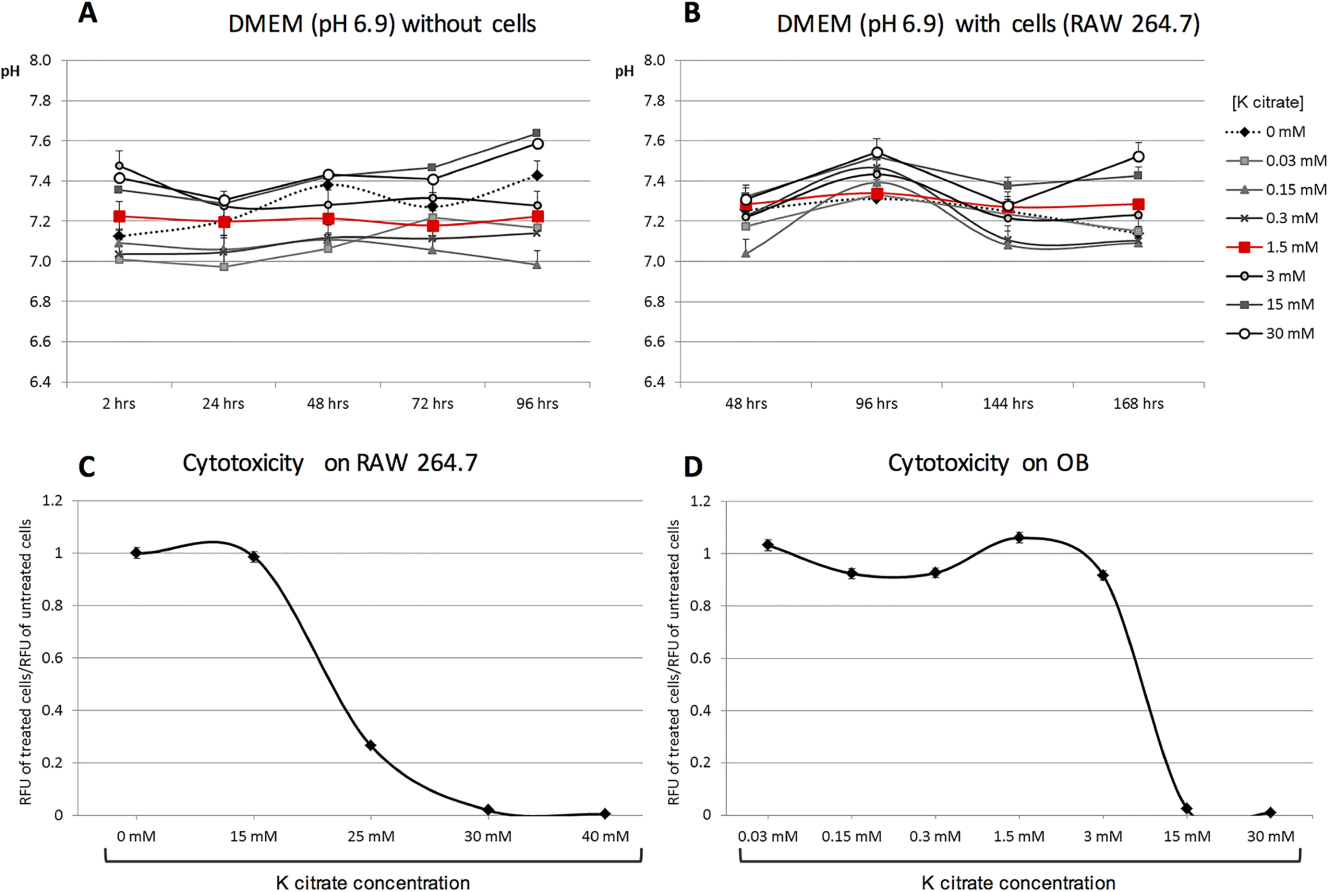
Alkalizing effect and cytotoxicity of K citrate. The **A** and **B** graphs represent the pH changes of acidic culture medium (pH 6.9) supplemented with different concentrations of K citrate (from 0.03 mM to 30 mM), in absence (**A**) and presence (**B**) of cells. In both graphs the red line highlights the concentration that raised pH steadily up to 96 hours. The **C** and **D** graphs represent the ratio between the RFU detected in cells cultures treated with different concentrations of K citrate and the RFU in untreated cultures (negative control, K citrate0 mM, ratio = 1). Results are expressed as mean ± SEM of three experiments. The IC50 at 96 hrs was 25.7 mM for RAW 264.7 cells (**C**) and 4.7 mM for OB (**D**).

To investigate the alkalizing capability of K citrate, we performed preliminary experiments using different K citrate concentrations in pH 6.8 culture media (15-25-30-40-50-100 mM), and evaluating the effect every 2 hours up to 10 hours, and after 24 hours. Values near pH 7.4 were achieved only with K citrate 50 and 100 mM, but the effect was transient and the doses were highly cytotoxic for RAW 264.7 cells (data not shown). We investigated the alkalizing capacity of K citrate using concentrations lower than 30 mM in culture media maintained at pH 6.9. The highest doses of K citrate (15 mM and 30 mM) were able to increase the pH value near to pH 7.4, but the effect was unstable during 96 hours. However, K citrate 1.5 mM increased pH from 6.9 up to 7.2 in culture medium alone (Fig 1A), and from 6.9 to 7.3 in presence of a continuous cell line of murine osteoclast progenitors, the RAW 264.7 cells (Fig 1B). Nevertheless K citrate 1.5 mM was sufficient to obtain a stable value of pH until 96 hours (Fig 1A and 1B). In the cytotoxicity screening, we used the RAW 264.7 cells, that were exposed to the acidic culture medium (pH 6.9) and K citrate concentrations ranging from 15 to 40 mM. As shown in Fig 1C, we found that the IC50 at 96 hours was 25.7 mM. In a second step the K citrate cytotoxicity was evaluated by using primary human OB cultured at pH 6.9 and challenged with K citrate concentrations ranging from 0.03 mM to 30 mM, and the IC50 at 96 hours was 4.7 mM (Fig 1D).

### K citrate inhibits the OC differentiation and activity promoted by the acidic milieu

**Fig 2:**
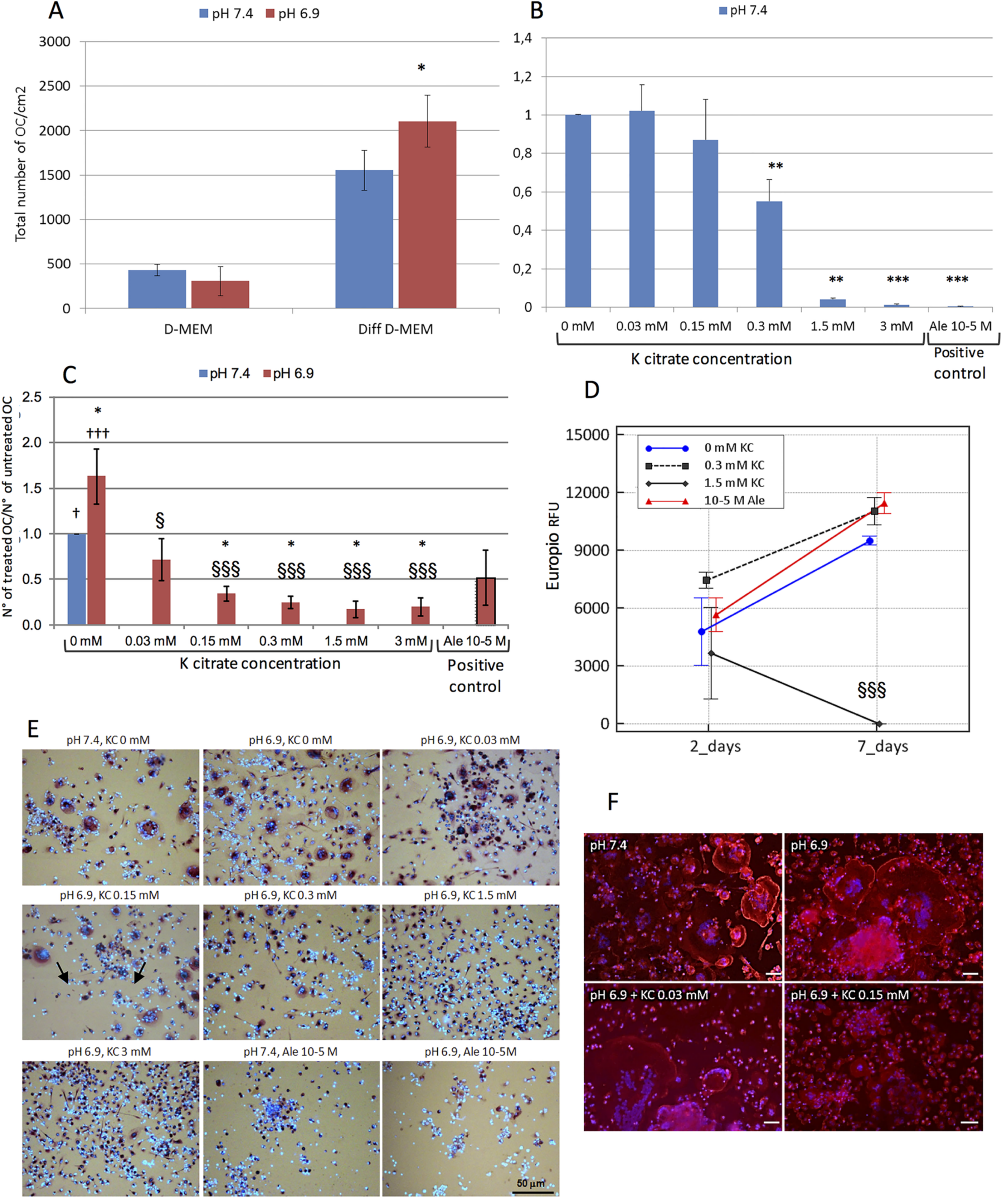
**A)** Effect of the acidic microenvironment on OC differentiation. Data are expressed as total number of OC/cm^2^. The bars represent the mean ± SEM of seven experiments performed at pH 7.4 and pH 6.9, **B)** K citrate inhibits the OC differentiation induced at pH 7.4. Results are expressed as a ratio between number of OC counted in cultures treated at pH 7.4 with different K citrate concentrations and number of OC counted in the negative control (pH 7.4, K citrate 0 mM, ratio = 1). Positive control is Alendronate 10^−5^ M. Mean ± SEM of four experiments. **C)** K citrate inhibits the OC differentiation induced by acidic microenvironment. Results are expressed as a ratio between number of OC counted in cultures treated at pH 6.9 with different K citrate concentrations and number of OC counted in the negative control (pH 7.4, K citrate 0 mM, ratio = 1). Mean ± SEM of six experiments. **D)** K citrate hampers the collagen degradation due to the activity of mature OC in acidic microenvironment. Results are expressed as mean ± SEM of RFU values of Europium-labeled collagen fragments in cultures of mature OC maintained at pH 6.9 (negative control) or treated with K citrate (KC). The experiment was repeated with cells obtained from two donors, with four replicates for each condition. **E)** OC are the cells with more than three nuclei (nuclear dye Hoechst 33258, blue) and a positive reaction for TRAP in the cytoplasm (red staining). The arrows indicate small and large OC. Magnification x 20, Scale bar = 50 μm. **F)** The phalloidin staining (TRITC, red) shows the typical dense belt-like structure of actin ring in multinucleated giant cells (blue nuclear-dye) cultured at pH 7.4 and pH 6.9; the ring microarchitecture is weakened after supplementation with K citrate 0.03 mM and no more recognizable with K citrate 1.5 mM. Magnification x 20, Scale bar = 50 μm. Symbols in histograms indicate statistically significant differences vs pH 7.4 negative control (*), vs Ale 10^−5^ M positive control (†), and vs K citrate 0 mM, pH 6.9 (§). P values ≤ 0.05, ≤ 0.01 and ≤ 0.001 were highlighted by one, two or three symbols, respectively.

Compared to neutral conditions, the acidic standard medium did not affect the spontaneous osteoclastogenesis of PBMC. However, under the influence of differentiating agents, the number of OC was significantly higher at pH 6.9 than at pH 7.4 (Fig 2A). Based on the IC50 calculated for OB cultures, we selected K citrate concentrations ranging from 0.03 mM to 3 mM to evaluate the impact of K citrate supplementation on OC differentiation and activity. As shown in Fig 2, the osteoclastogenesis was significantly inhibited by K citrate in a dose-dependent manner and irrespective of neutral (Fig 2B) or acidic (Fig 2C and 2E) conditions. In particular, the strongest anti-osteoclastogenic effect was observed with 1.5 mM and 3 mM K citrate. The OC cultured at pH 7.4 exhibited the typical dense belt-like structure of actin which is essential for bone resorption (Fig 2, pH 7.4). The acidic microenvironment enlarges the osteoclast size but does not affect the actin ring (Fig 2, pH 6.9). The lowest doses of K citrate induced a partial disruption of ring formation (Fig 2, KC 0.03),while it was no longer perceivable when the inhibition of osteoclastogenesis became evident (Fig 2, KC 0.15). We also evaluated the effects of K citrate on bone resorption by using K citrate 0.3 mM and 1.5 mM. PBMC were induced to differentiate on a collagen surface for seven days, and after completing the differentiation process, the mature OC were exposed to acidic medium supplemented with K citrate or Alendronate. The degradation of Type I collagen was measured after 2 and 7 days and it was hampered significantly when mature OC were treated with K citrate 1.5 mM. Noteworthy, the anti-resorptive properties of Alendronate were lacking when mature OC were maintained at pH 6.9 (Fig 2D).

**Fig 3:**
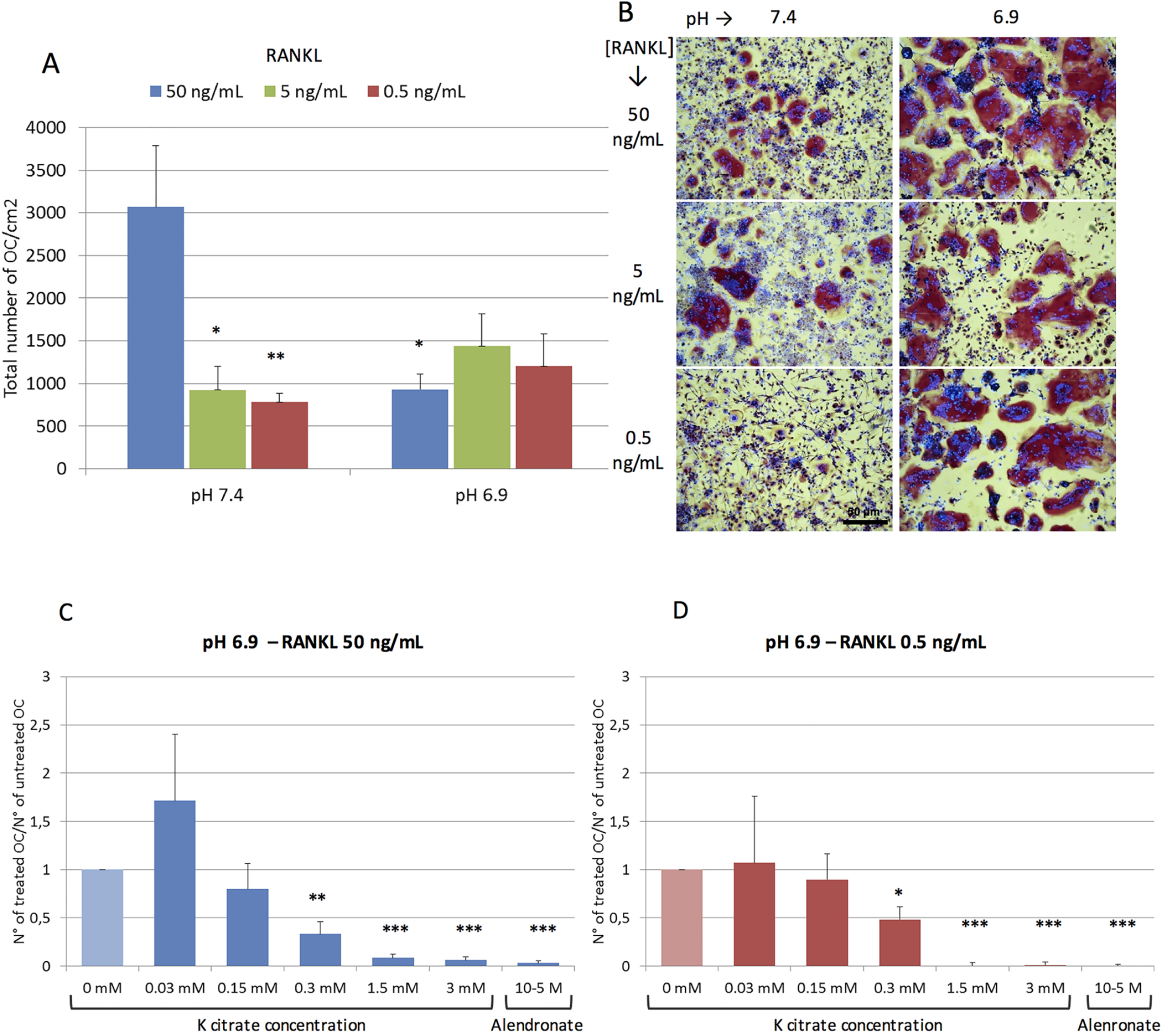
**A)** RANKL-mediated OC differentiation in acidic and neutral microenvironment. The bars represent the mean ± SEM of the total number of OC/cm^2^ obtained from buffy-coat of four individuals and cultured at pH 7.4 and pH 6.9 with three different RANKL concentrations. Symbols (*) indicate statistically significant differences vs RANKL 50 ng/mL at pH 7.4. **B)** The representative pictures show that the size of TRAcP positive multinucleated cells and the differentiating potency of low RANKL concentrations are higher at pH 6.9 than at pH 7.4. Magnification x 10, Scale bar = 50 μm. **(C)** and **(D)** K citrate inhibits the OC differentiation induced at pH 6.9 by high (C: 50 ng/mL) and low (D: 0.5 ng/mL) RANKL doses. Results are expressed as a ratio between the number of OC counted in cultures treated with different K citrate concentrations and number of OC counted in the negative control (K citrate 0 mM, ratio = 1). The positive control is Alendronate 10^-5^M. Mean ± SEM of data obtained from three different donors and two replicates for each condition. Symbols (*) indicate statistically significant differences vs negative control (pH 6.9, K citrate 0 mM, ratio = 1). In all histograms P values ≤ 0.05, ≤ 0.01 and ≤ 0.001 were highlighted by one, two or three symbols, respectively.

The OC differentiation was evaluated either using the conditioned medium containing a cocktail of differentiating factors, or growing the OC progenitors with specific recombinant proteins, i.e. RANKL and M-CSF (Fig 3). Although RANKL 50 ng/mL favored the OC differentiation in all experiments performed at pH 7.4, the number of TRAcP positive multinucleated cells obtained at pH 6.9 varied among individuals (Fig 3A). Nevertheless, at pH 6.9 the OC size was larger than that observed at pH 7.4, thus suggesting that the acidic microenvironment could promote the fusion of OC progenitors (Fig 3B). Furthermore, in neutral conditions we observed that the OC number decreased significantly when the RANKL dose moved from 50 ng/mL to 0.5 ng/mL, while no significant differences were observed at pH 6.9 (Fig 3A and 3B). K citrate was able to hamper effectively the OC differentiation in presence of the highest and lowest RANKL concentrations (Fig 3C and 3D).

**Fig 4:**
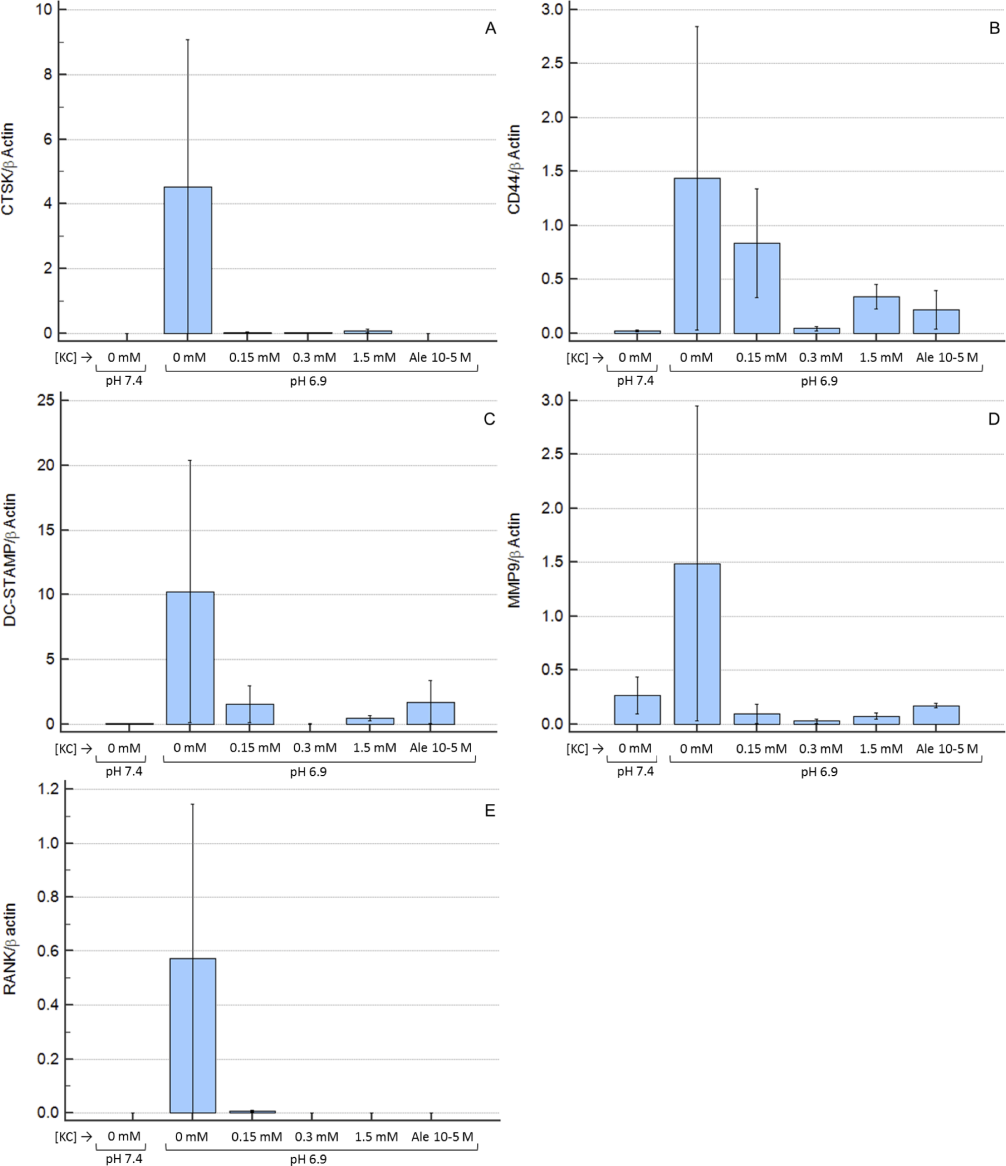
Expression of genes involved in RANKL-mediated OC differentiation. Results are expressed as ratio between the expression of “gene of interest” and “β-actin” as housekeeping gene. Mean ± SEM of data obtained from three technical replicates for each condition. **A**: Cathepsin K, **B**: CD44, **C**: DC-STAMP, **D**: MMP-9, **E**: RANK. KC is K citrate.

At day 14, the expression of genes involved in RANKL-mediated OC differentiation was higher at pH 6.9 in comparison to pH 7.4, and the K citrate supplementation of the acidic culture medium was able to down-regulate the transcription of RANK, CD44, DC-STAMP, Cathepsin K and MMP-9 until levels observed in neutral conditions (Fig 4A and 4E). No statistically significant difference was detectable since molecular analyses showed a high variability.

### K citrate overcomes the resistance to alendronate promoted by the acidic milieu

**Fig 5:**
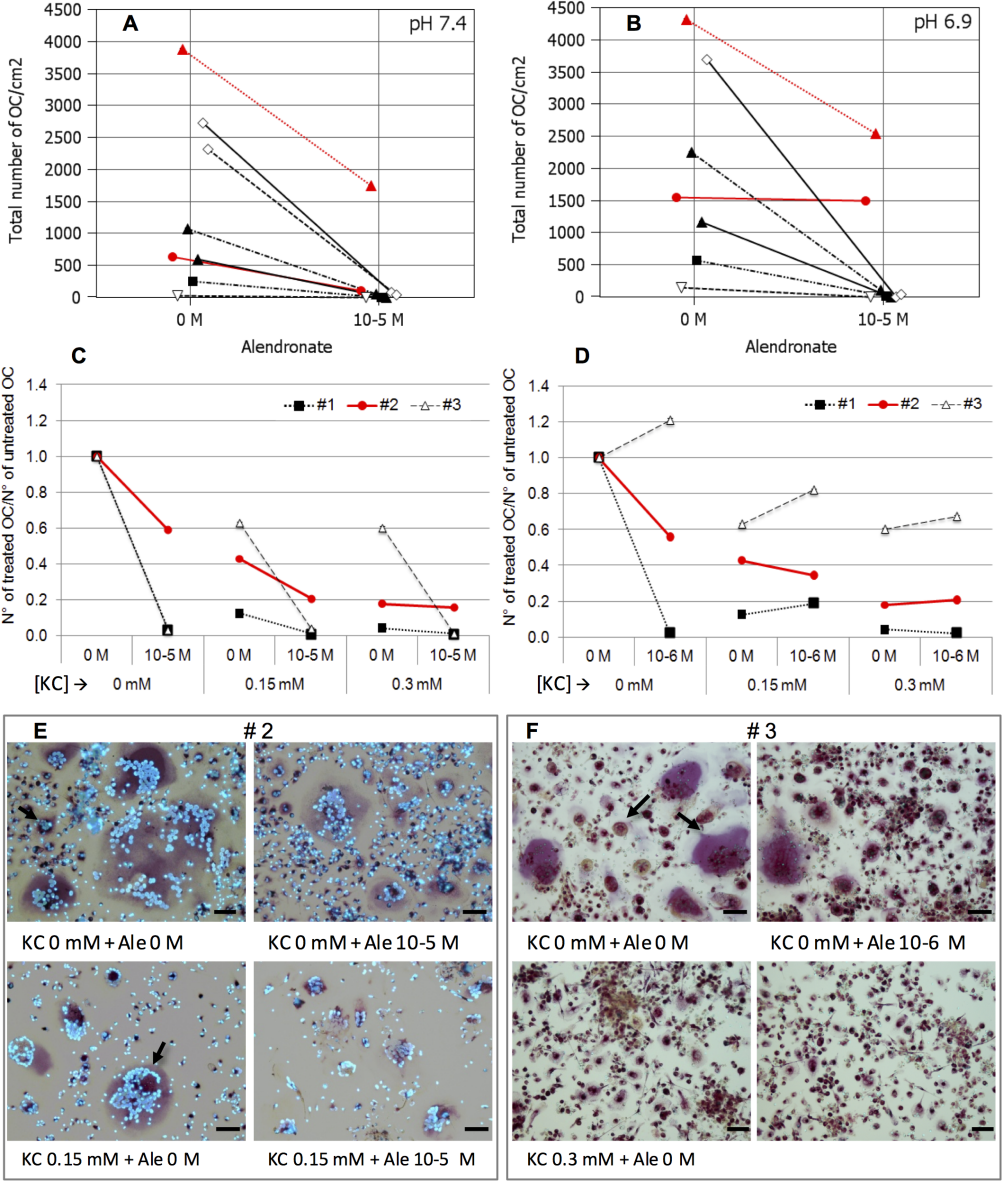
Effect of K citrate on OC cultures resistant to alendronate under the effect of acidic microenvironment. Data are expressed as total number of OC counted in eight separate experiments. The OC cultures react differently to the alendronate in neutral (**A**) or acidic (**B**) culture medium. The red lines highlight two cultures which were resistant to the anti-osteoclastogenic effect of Ale 10^−5^. The **C** and **D** graphs show the data of three separate experiments (#1; #2; #3) exposed to Ale 10^−5^ (**C**) and Ale 10^−6^ (**D**), supplemented with K citrate (0 mM, 0.15 mM, 0.3 mM). Results are expressed as a ratio between number of OC treated with different concentrations of K citrate/ alendronate, and number of OC counted in the negative control, i.e. K citrate 0 mM, Ale 0 mM, pH 6.9. The red line is the OC culture resistant to Ale 10^−5^ (#2), and representative images are shown in the panel **E**. The dashed line is the OC culture which is stimulated by Ale 10^−6^ (#3), and the pictures below highlight the effect of K citrate (**F**). OC are the cells with more than three nuclei and red staining of cytoplasm (positive reaction to TRAP). The arrows indicate small and large OC. KC is K citrate. Magnification x 20, Scale bar = 50 μm.

We observed that the pharmacological activity of bisphosphonate varied among donors and was influenced by pH in culture medium. As expected, in six out of eight OC cultures, alendronate significantly inhibited osteoclastogenesis both at pH 6.9 or pH 7.4. However, in one case the OC number was so high that at both pH conditions Ale 10^−5^ M only partially prevented OC formation (Fig 5A), and in one OC culture Ale 10^−5^ was completely ineffective at pH 6.9 (Fig 5B). Therefore, we evaluated if the combination of alendronate with K citrate enhanced the anti-osteoclastogenic effect of both compounds. As shown in Fig 5C, 5D and 5K citrate was able to potentiate the bisphosphonate activity. Indeed, K citrate 0.15 mM reduced the OC number up to 60% while the maximum obtained with Ale 10^−5^ was 40%. Moreover, we observed a synergy between K citrate and Ale 10^−5^ since the combination of the two compounds inhibited the 80% of OC formation. K citrate 0.3 mM inhibited the osteoclastogenesis at the same extent, but the combination with Ale 10^−5^ did not increase the pharmacologic effect (Fig 5C). The synergistic effect was also evaluated with a lower concentration of alendronate (Fig 5D). We found that Ale 10^−6^ promoted the OC differentiation in one cell culture totally responsive to Ale 10^−5^. In this case, K citrate 0.15 mM decreased the OC number up to 60%, while the combination with Ale 10^−6^ restored the drug resistance partially. Once again, no additional anti-osteoclastogenic effect was observed when K citrate 0.3 mM and Ale 10^−6^ were combined.

### K citrate is unable to fully restore the OB function altered by the acidic milieu, but influences proliferation and alkaline phosphatase expression

**Fig 6:**
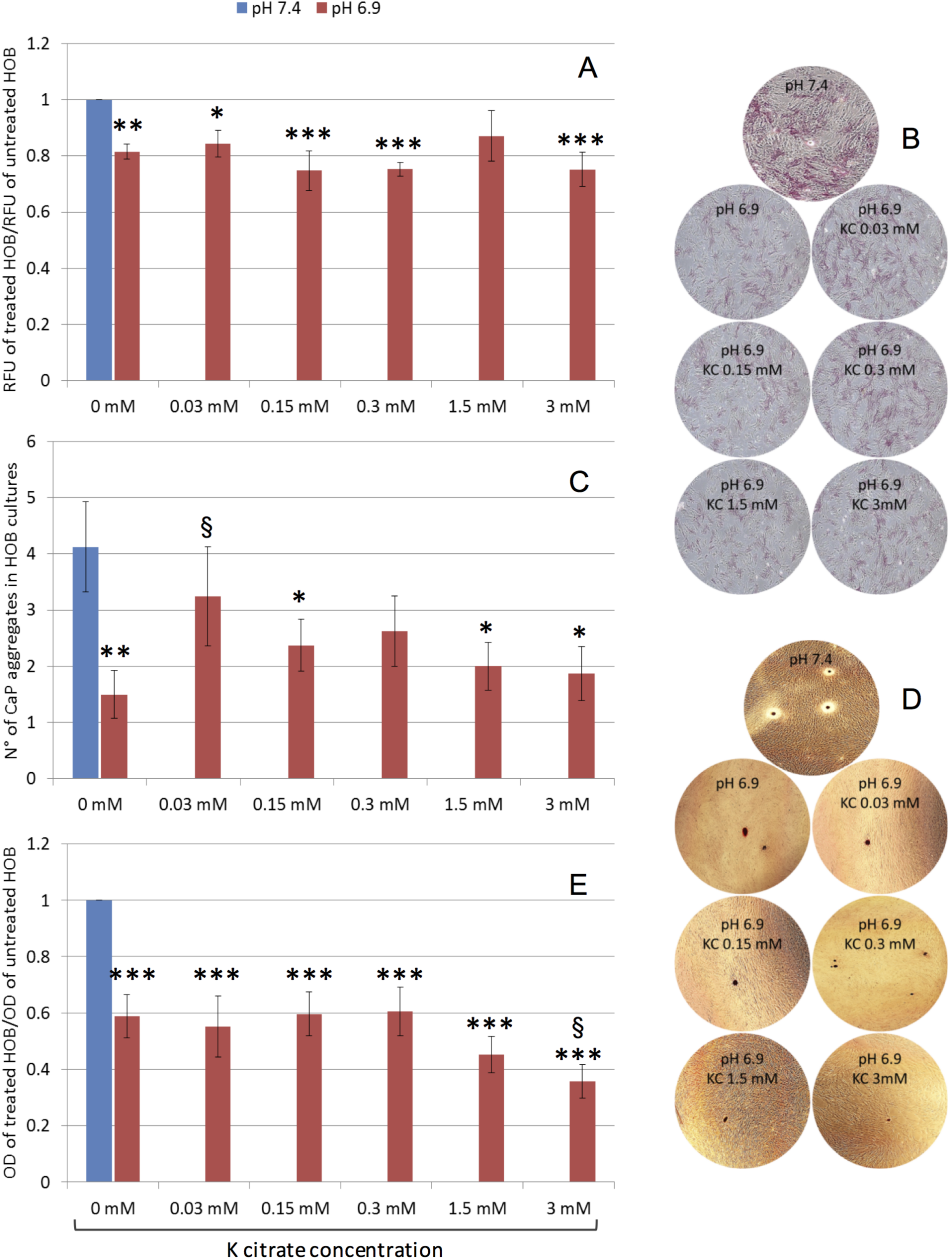
Effect of K citrate on OB cultures grown in acidic milieu. **A)** Cell viability of OB treated with K citrate in acidic microenvironment (pH 6.9) and comparison with OB cultured in buffered medium (pH 7.4). Results are expressed as a ratio between the RFU detected in cultures treated with different concentrations of K citrate (from 0 mM to 3 mM) and the RFU in untreated cultures maintained at pH 7.4 (negative control, ratio = 1). The bars represent mean ± SEM of five experiments. **B)** Representative images of cytochemical activity of alkaline phosphatase (red staining). Magnification x 4. **C)** Mineral nodules formation. Results are expressed as number of calcium phosphate aggregates stained with Alizarin Red dye. **D)** Representative images of mineral nodules (Alizarin Red staining, red) Magnification x 4. **E)** The elution of Alizarin Red dye is expressed as a ratio between the OD of the solubilized dye in cultures treated with K citrate and in negative control. In **C)** and **E),** the bars represent mean ± SEM of four experiments, in duplicate. In all histograms, symbols indicate statistically significant differences vs negative control (*) and vs K citrate 0 mM, pH 6.9 (§). One, two or three symbols indicate P values ≤ 0.05, ≤ 0.01 and ≤ 0.001, respectively.

The same conditions used for OC cultures were applied to investigate the effect of K citrate on OB maintained in acidic culture medium. We found that cell proliferation was significantly decreased at pH 6.9 compared to the physiologic pH (Fig 6A). K citrate did not further inhibit cell growth, and with 1.5 mM the proliferation rate was partly restored as observed at neutral pH.

Extracellular acidosis strongly reduced the mineralization ability of bone-forming cells. Indeed, a decrease cytochemical activity of alkaline phosphatase (Fig 6B) and a significantly lower number of CaP nodules were observed in OB cultures maintained at pH 6.9 (Fig 6C and 6D). However, the different concentrations of K citrate were ineffective to restore the proper mineralization process, as confirmed by quantifying the elution of the Alizarin Red (Fig 6E). Also, the highest K citrate concentration seemed to hamper the mineralization since the OD of the eluted dye was significantly decreased in comparison to the untreated cultures.

**Fig 7:**
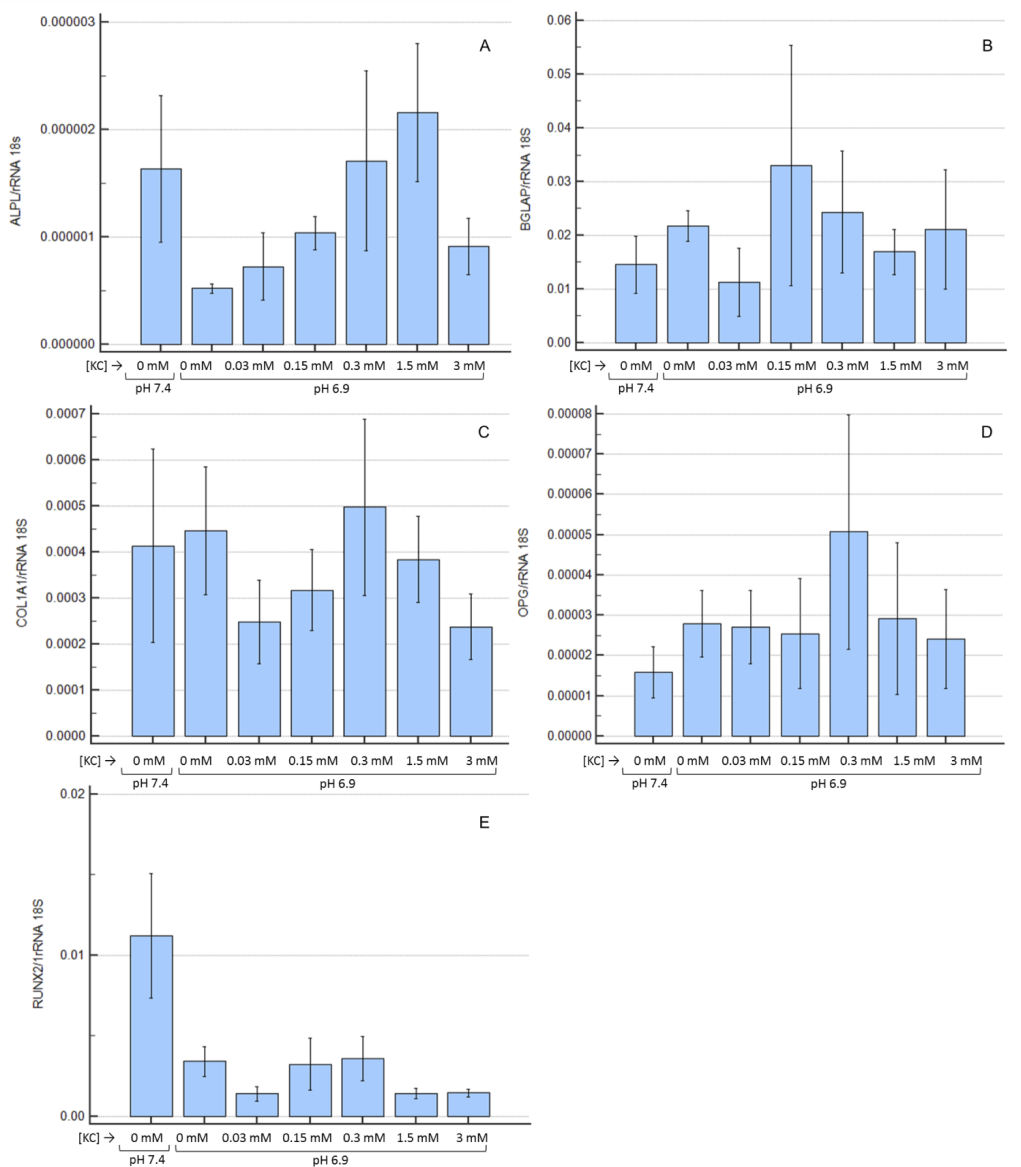
Expression of bone-related genes in OB cultured at pH 6.9 and treated with K citrate. Results are expressed as a ratio (log_10_ transformed) between the expression of ALPL (**A**), BGLAP (**B**), COL1A1 (**C**), OPG (**D**) and Runx2 (**E**) in cultures treated with different concentrations of K citrate and the expression in negative control (K citrate 0 mM; pH 7.4; ratio = 1). The bars represent mean ± SEM of three experiments in duplicate.

Not only the enzyme activity, but also ALPL expression was lower in OB maintained at pH 6.9 (Fig 7A), but K citrate, from 0.15 mM to 1.5 mM, up-regulated the transcript level of ALPL in a dose-dependent manner. The expression of BGLAP (Fig 7B) and COL1A1 (Fig 7C) was highly variable. The OPG expression (Fig 7D) was enhanced at pH 6.9, and K citrate 0.3 mM further increased the transcript level. Runx2 (Fig 7E) was down-regulated in presence of extracellular acidosis, and no inverse trend was determined by K citrate. Finally, in most cultures Osterix and RANKL expression was under the detection limits, and treatment with K citrate did not modify the transcript levels (data not shown). In spite of the above-mentioned trends, the experimental variability did not allow to highlight statistically significant differences.

## Discussion

The excessive acid load is physiologically balanced through metabolic adaptation that involves primarily kidney, lung, and bone [28]. In bone tissue, the acidic microenvironment affects bone cells and favors the resorption of the mineralized matrix in order to recruit hydroxyl groups that neutralize the proton excess when the capacity of other buffer systems is limited [4]. However, if the acidic overload persists, the bone becomes osteopenic due to the net loss of calcium and deterioration of microarchitecture. The oral administration of alkali compounds, i.e. K citrate in doses ranging from 30 to 90 mmol/day, has been proposed for opposing the low-grade acidosis and preventing bone loss [15,16,18–22]. Although preliminary clinical data are encouraging, it is still unclear whether the beneficial effects are exclusively due to the alkalizing function of K citrate, or if it directly influences bone cell activity. In this study, we aimed to investigate if K citrate is able to counteract the adverse effects induced by acidosis regarding the resorption activity of OC and the mineralization capability of OB.

To mimic a low pH milieu, we used culture media buffered up to pH 6.8 and pH 6.9. We observed that only very high concentrations of K citrate were able to neutralize pH 6.8, but these doses were cytotoxic and the alkalizing effect disappeared after 24 hours (data not shown). The pH 6.9 was neutralized with doses higher than 3 mM, but monitoring the cell-free culture medium and the RAW 264.7 supernatant until 168 hours we found that the alkalinization did not persist over time, even if a quite stable condition was obtained with K citrate 1.5 mM. Since a number of studies have shown that the detrimental effects of acidosis on OB and OC were detectable at pH 6.9 [4,5,10,29], we chose this pH condition for culturing bone cells.

Our first goal was to select K citrate doses to be used in the experimental setting. Firstly, we excluded K citrate concentrations highly cytotoxic for RAW 264.7, a murine cell line which exhibits biological features similar to those of OC precursors [30]. Then, since bone-forming cells are the cellular components to be preserved, the cytotoxicity screening was refined on human OB maintained at pH 6.9. Having defined the non-cytotoxic doses, we evaluated the effects of K citrate on primary human bone cells cultured in acidic microenvironment.

A spontaneous osteoclastogenesis has been observed in all cultures without differentiating stimuli [31], but it was not influenced by the acidity of culture medium. On the contrary, the low pH fostered the activity of pro-osteoclastogenic factors and promoted the OC differentiation. Although the positive effects of acidosis on OC function have been widely described by other Authors [5,10,29,32–34], our study shows a noteworthy result, that is the significant osteoclastogenesis inhibition induced by non-cytotoxic doses of K citrate. In particular, K citrate 1.5 mM not only shifted the pH from 6.9 to 7.2 effectively over time but significantly hampered the differentiation of OC precursors and the collagen degradation by mature OC. Since K citrate was effective also at pH 7.4, we may exclude that it could inhibit only the OC activity triggered by the acidic microenvironment. Conversely, it is reasonable to hypothesize that the anti-osteoclastogenic potency depends on something which is not necessarily related to alkalinizing properties of the compound.

To obtain the OC differentiation, we used a conditioned medium derived from a continuous cell line whose efficacy in promoting the osteoclastogenesis has been previously shown [23,35–37]. In order to clarify the mechanisms involved in K citrate activity, the experiments have been repeated using the specific recombinant proteins that are considered as essential factors in driving the osteoclastogenesis, i.e. RANKL and M-CSF [38]. Indeed, M-CSF promotes the expression of RANK, that is the RANKL receptor RANK, Then, the RANKL/RANK complex initiates the differentiation of OC precursors, promotes their fusion into mature OC and enhances osteoclast survival and activity [38].

The RANKL level of the conditioned medium used for our experimental setting was lower than 2 ng/mL [23]. Since it was used at 20%, the amount available for OC differentiation wasfar from that used in current culture methods (20–100 ng/ml) [39], but closer to the circulating levels measured in disorders of bone remodeling (0.5–1 pmol/L, unit conversion: 1 pg/ml = 0.05 pmol/L) [40]. In the experiments based on the use of recombinant proteins, high RANKL concentrations (50 ng/mL) allowed to obtain an effective OC differentiation at neutral pH, while in acidic microenvironment the response was less pronounced in terms of OC number. However, in spite of the highest OC number at pH 7.4, the low pH fostered the fusion of precursors, as proved by the large size of multinucleated TRAcP positive cells. Unexpectedly, the extracellular acidosis favored the OC differentiation even in the presence of low RANKL doses which are ineffective at neutral pH. In the field of bone remodeling pathophysiology, this is a relevant finding since suggests that low RANKL levels combined with a clinical sub-acidosis are sufficient to sustain a decrease in bone mass.

The anti-osteoclastogenic activity of K citrate could depend on a variety of mechanisms. Other Authors demonstrated that extracellular acidosis mirrors RANKL activity, thus promoting the nuclear accumulation of "nuclear factor-activated T cells c1" (NFATc1) in OC precursors and activating the Ca^2+^/calcineurin/NFATc1 pathway and specific genes which play a key role in OC differentiation [41,42]. In fact, the low pH enhanced the expression of genes critical for osteoclastic cell-cell fusion, i.e. DC-STAMP [43]. Other genes strictly related to OC differentiation and activity were overexpressed at pH 6.9 compared to pH 7.4, and were down-regulated in presence of K citrate, including RANK or RANKL receptor [38], CD44 which plays a crucial role in podosome organization [44], and CTSK and MMP-9 which are two enzymes deputed to the degradation of mineralized bone matrix [45]. Since all the above genes are down-regulated in presence of K citrate, it is reasonable to hypothesize that the partial neutralization obtained with the compound may hamper osteoclastogenesis through the “switch off” of the Ca^2+^/calcineurin/NFATc1 pathway. The alkalizing activity of K citrate might reduce the activation of the acid-sensing receptors identified on the plasma membrane of OC, including ovarian cancer G-protein-coupled receptor 1 (OGR1) [46,47], T cell death-associated gene 8 (TDAG8) [48], transient receptor potential (TRP) family members [31,33], and the acid-sensitive ion channel (ASIC) subunits [42,49]. All the above mentioned molecules increase intracellular Ca^2+^, thus favoring the NFATc1 activation and OC differentiation [34].

A remarkable inhibition of OC formation and activity occurred with K citrate 1.5 mM, which determined a pH increase up to 7.2 persisting over time. Consistent with our findings, Arnett *et al*. have reported that pH 7.2 acts as a "switch on" to initiate bone resorption, and just below this value the OC activity increases significantly until a plateau is reached at pH 6.9 [10]. However, the osteoclastogenesis inhibition is also observed with low K citrate concentrations which are unable to modify the pH of the culture medium. Probably, the pharmacological properties of this compound do not exclusively depend on the alkalizing capacity, but also the chemistry could play a pivotal role. Both potassium and citrate regulate a variety of bone cell functions, and, in clinical practice, their biological activities could potentiate the benefits deriving from K citrate as a tool for preventing the osteopenia progression. Recently, we evaluated some parameters of lithogenic risk related to the acid-base balance, and we observed that, other than low pH, also the low excretion of citrate and potassium is a common finding in osteopenic postmenopausal women without concomitant diseases [17]. Therefore, the alkali supplementation is expected to be effective in opposing the acid load excess, but K citrate may be more efficient since it provides two elements that play a remarkable role in bone physiology. Some authors have demonstrated that the increase of extracellular K^+^ inhibits the RANKL-induced OC differentiation in a dose-dependent manner, and the anti-osteoclastogenic activity is mediated by a potassium channel subfamily that is considered as a negative regulator of OC differentiation [50]. Citrate is a primary product of the mitochondrial oxidative metabolism, and high citrate concentration in cytosol has an inhibitory effect on the activity of phosphofructokinase, a rate-limiting enzyme of glycolysis [51]. Therefore, citrate supplementation yields a negative effect on the glycolytic pathway, and changes in the energy systems affect differentiation and function of OC [52,53]. Finally, citrate owns a calcium-binding capacity, and therefore it may reduce the Ca^2+^ availability, thus interrupting the NFATc1 signaling activated by the proton excess [33,34]. Similarly, other Authors have shown that intracellular Ca^2+^ chelation hampers the OC survival induced by RANKL [54].

As previously mentioned, K citrate inhibited osteoclastogenesis as much as Ale ^10–5^, which is routinely used as a positive control for the *in vitro* screening of anti-resorptive compounds [25]. Nitrogen-containing bisphosphonates, including alendronate, inhibit a key enzyme in the post-translational events regulating the intracellular signaling of OC [55]. Our results suggest that K citrate and alendronate do not share the same pharmacological activities since in at least one case the anti-osteoclastogenic effect of Ale was abrogated at pH 6.9 while K citrate was effective. We can translate these results in clinical setting, thus hypothesizing that a low-grade acidosis could play a role in determining the bisphosphonate-unresponsiveness that is observed in a proportion of patients [56]. In this context, the alkalizing supplementation may support the conventional therapy of the osteoporosis and could potentiate the effect of bisphosphonate.

We observed a direct effect of acidosis on bone-forming cells, and particularly proton excess prevents the formation of mineral nodules in OB cultures. By itself, the acidic milieu favors the dissolution of bone matrix [11], but, also, it may alter some cell functions involved in the mineralization process. In agreement with literature data, we found that pH 6.9 decreases ALPL and Runx2 expression [57], induces a slight increase in OPG, while collagen and BGLAP were not affected [5,58,59]. Alkaline phosphatase plays a pivotal role in regulating bone mineralization, since it catalyzes the inorganic pyrophosphate (PPi) hydrolysis and provides inorganic phosphate (Pi) for the nucleation of hydroxyapatite crystals [60]. Orriss et al. have demonstrated that acidosis affects the PPi/Pi balance by decreasing ALPL and promoting the expression of the enzyme involved in PPi generation [61]. However, K citrate was unable to fully restore the bone-forming ability of OB. Even though citrate has been identified as an essential component of the apatite/collagen structure [62], the OB capability to form mineral deposits does not improve if the acidic medium is supplemented with K citrate. As the nontoxic concentrations of K citrate are not sufficient to raise the pH up to 7.4, we can speculate that the proton excess in culture medium is the main responsible for the altered cell function with regard to the mineralization. On the contrary, the pharmacological activity of potassium and citrate that we considered to explain the positive effects on OC cultures seems to be less relevant.

## Conclusions

In summary, this study has been planned to highlight the K citrate activity on biological epiphenomena regarding bone cells, i.e. the formation of new OC, the mineralized matrix resorption, and the mineralized matrix deposition. For the first time, we showed that K citrate inhibits osteoclastogenesis and enhance the anti-osteoclastogenic activity of alendronate, and that such effect does not depend exclusively on its alkalizing properties. Our results provide a biological basis for the use of K citrate in preventing the bone loss that occurs in low-grade acidosis, and offer a number of suggestions to explore deeply the anti-osteoclastogenic role of K citrate in clinical setting. Finally, the anti-resorptive properties of K citrate have been demonstrated also at low doses, but clinical studies are needed to define dosage schedule, timing and duration of supplementation, and to determine the effective benefits of this strategy in preventing the osteopenia progression.

## Supporting information

S1 File OC number raw data of Fig 2. (XLSX)

S2 File OC number raw data whit RANKL of Fig 3. (XLS)

S3 File OC gene expression raw data of Fig 4. (XLS)

S4 File Alendronate treatment raw data of Fig 5. (XLS)

S5 File OB proliferation raw data of Fig 6. (XLS)

S6 File OB gene expression raw data of Fig 7. (XLS)
